# Prognostic Significance of p53 and p63 in Diffuse Large B-Cell Lymphoma: A Single-Institution Experience

**DOI:** 10.3390/curroncol30020102

**Published:** 2023-01-17

**Authors:** Juan Carlos Alvarez Moreno, Hisham F. Bahmad, Abed Alhalim Aljamal, Ruben Delgado, Ali Salami, Carolina Guillot, Amilcar A. Castellano-Sánchez, Ana Maria Medina, Vathany Sriganeshan

**Affiliations:** 1 Arkadi M. Rywlin M.D. Department of Pathology and Laboratory Medicine, Mount Sinai Medical Center, Miami Beach, FL 33140, USA; 2 Department of Internal Medicine, Mount Sinai Medical Center, Miami Beach, FL 33140, USA; 3 Department of Mathematics, Faculty of Sciences, Lebanese University, Nabatieh 1700, Lebanon; 4 Department of Translational Medicine, Herbert Wertheim College of Medicine, Florida International University, Miami, FL 33199, USA

**Keywords:** p63, p53, Ki-67, DLBCL, prognosis, overall survival, progression-free survival, double expressors

## Abstract

Diffuse large B-cell lymphoma (DLBCL) is the most common lymphoma in adults. We evaluated the immunohistochemical (IHC) expression of p63 and p53 in DLBCL and their significance on overall survival (OS) and progression-free survival (PFS). We conducted a retrospective cohort study of 177 patients with DLBCL who presented to Mount Sinai Medical Center of Florida (Miami Beach, Florida) between 2010 and 2020. IHC staining for p63 and p53 protein expression was performed. A significant correlation was found between p63 positivity and p53 expression, p53/p63 co-positivity, Ki-67 proliferation index, MYC expression, and MYC/BCL2 double expression. Regardless of the germinal center B-cell like (GCB) subgrouping, there was a trend among p53+ patients to have MYC/BCL2 double expression, positive MYC expression, and lower OS and PFS. A tendency of poor OS was seen in p53^+^ patients in the non-GCB, GCB, and double expressors subgroups and poor PFS in p53^+^ patients regardless of the subgrouping. In conclusion, our results suggest that p63 and p53 may represent potential additional prognostic biomarkers in DLBCL and may be included in the initial diagnostic work up of patients with DLBCL.

## 1. Introduction

Diffuse large B-cell lymphoma (DLBCL) is the most common lymphoma in adults, accounting for 30–40% of all non-Hodgkin lymphomas (NHL) in Western countries [1,2]. In the United States, the annual incidence is 19.6 cases per 100,000 person-years for both men and women [3]. The World Health Organization (WHO) defines DLBCL as a biologically heterogeneous neoplasm with diverse prognosis. The 5-year post-treatment survival is approximated to be 78% [4].

This tumor may arise de novo or as a transformation from other low-grade lymphomas. The clinical presentation is usually an enlarging mass at single or multiple lymph nodes or extranodal sites. Microscopically, the lymph node or extra nodal site is completely or partially replaced in most cases by sheets of medium-sized to large lymphoid cells with increased proliferation rate. Morphological, biological, and clinical studies have assigned multiple classification schemes to DLBCL. The most widely used classification system is based on the cell of origin as germinal center B-cell like (GCB) type and non-GCB type [5]. The standard of care therapy for the initial treatment includes rituximab, cyclophosphamide, doxorubicin, vincristine, and prednisone (RCHOP-21) every 21 days (3-week cycle) [6,7]. In addition, ongoing research using new drugs to target oncogenic pathways are in progress [8]. The difference in survival due to the biologic heterogeneity of these neoplasms has led to extensive investigation on additional prognostic stratification of these tumors. In this study, we explored two potential biomarkers, p53 and p63, to provide an expanded prognostic profile of these neoplasms.

The *p63* gene located on chromosome 3q27-28 is a member of the *p53* gene family; it encodes for the p63 protein, which is expressed in the nuclei of epithelial cells of stratified epithelia as well as basal cells in glands of the prostate, breast, and bronchi [9]. In non-epithelial tissue, such as lymph nodes, p63 has been shown to be expressed in germinal center cells [10]. There are two isoforms of p63, the TA form that contains a transactivation domain with 22% homology to its counterpart p53, and the N form that lacks the N-terminal domain [11,12]. These two isoforms regulate and antagonize p53 working as either tumor suppressors or oncogenes [10]. The TA form of p63 is expressed in the germinal center cells, specifically in B-lymphocytes, showing positivity in some B-cell lymphomas [9].

There are currently conflicting conclusions on the role of p63 as a prognostic marker in lymphomas. While studies by Fukushima et al. [13] and Hu et al. [14] showed a poor prognosis in DLBCL, others including Xu-Monette et al. [15] and Hallack Neto et al. [16] showed improved survival in lymphomas highly expressing p63. In our study, we evaluated the immunohistochemical (IHC) expression of p63 and p53 in DLBCL and correlated between the level of expression and other clinicopathological parameters to determine their effect on overall and progression free survival.

## 2. Materials and Methods

### 2.1. Study Design and Setting

We conducted a retrospective cohort study of 177 patients with DLBCL who presented to Mount Sinai Medical Center (MSMC, Miami Beach, FL, USA) over a period of ten years, from 1 January 2010, until 31 December 2020. Inclusion criteria encompassed patients diagnosed with DLBCL at MSMC. Exclusion criteria included: patients with (1) co-existing carcinoma or sarcoma, (2) neoadjuvant therapy prior to the diagnosis of DLBCL, (3) co-existing or prior lymphomas other than DLBCL (for example, Hodgkin lymphoma), and (4) double/triple hit lymphoma (*MYC*, *BCL2*, and *BCL6*). Clinicopathological parameters of patients who met the inclusion criteria were retrieved from electronic medical records. The variables collected included age, gender, lactate dehydrogenase levels (LDH), clinical stage, response to therapy (recurrence, remission, or death), overall survival (OS; in months), progression-free survival (PFS; in months), presence of extranodal disease at the time of diagnosis, subtypes (GCB vs. non-GCB) using the Hans algorithm [17], high versus low Ki-67 expression (using a cut off of 80% as previously described in other studies [18]), flow-cytometry results (monoclonal B-cell population vs. negative), *MYC* expression, *BCL2* expression, and double expressor status. OS was calculated from the date of diagnosis to the date of last follow-up or death, whereas PFS was calculated from the date of diagnosis to the date of disease progression or death [15].

### 2.2. Histopathologic and Immunohistochemical Staining

Tissue samples were fixed in neutral buffered formalin and embedded in paraffin for histologic processing. Slides of the lymphoid and non-lymphoid tissue sections with DLBCL were stained with p63 and p53 immunohistochemistry (IHC). The corresponding Hematoxylin & Eosin (H&E) slides were analyzed in accordance with their compatible IHC stains. 

IHC staining for p63 and p53 (Ventana Medical Systems Inc., Tucson, AZ, USA) protein expression was performed in sections cut at 4 µm from formalin-fixed, paraffin-embedded (FFPE) tissue blocks and using the automated IHC protocols by Ventana Medical Systems. Appropriate positive and negative controls were used. The expression was interpreted as positive when the lymphoma cells showed golden-brown nuclear staining and negative when there was no staining. Screening of the lymphoid and non-lymphoid tissues was performed in a systematic manner. Areas with positive stain in epithelial and non-epithelial tissue were avoided when selecting hot-spots of the lymphoma cells. 

### 2.3. Histopathologic and Immunohistochemical Evaluation

**Figure 1:**
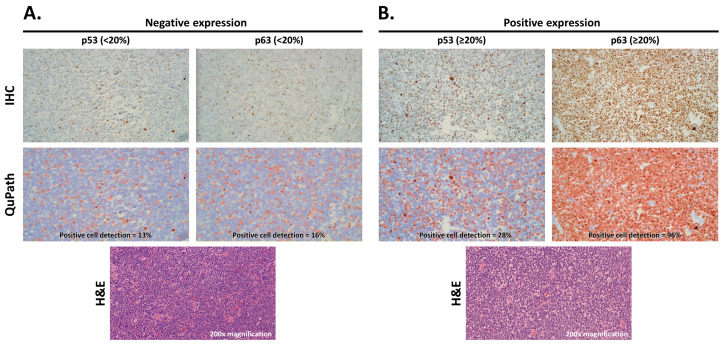
Representative H&E images, IHC of p53 and p63 staining, and positive cell detection using QuPath images showing negative expression (**A**) and positive expression (**B**). Negative expression (<20%) is depicted as blue nuclei and positive expression (≥20%) as red nuclei in QuPath (200× magnification).

Pictures were taken at 200× magnification with a digital microscope camera with hot-spot areas of p63 and p53 staining. QuPath (open-source software; version 0.2.3) for bioimage analysis [19,20] was used to quantify p63 and p53 stains (Figure 1). Percentage of p53 or p63 positivity was calculated by counting lymphoma cells with positive staining over the total number of lymphoma cells. Areas of positive non-lymphoma cells were excluded from the image analysis. p63 and p53 expression was categorized into less than 20% and greater than or equal to 20%. 

### 2.4. Statistical Analysis

Data retrieved from electronic medical records was entered into a Microsoft Excel spreadsheet designed for this study and then transferred into the Statistical Package of Social Science (IBM Corp., Released 2013, SPSS Statistics for Windows Version 22.0, Armonk, NY, USA). Quantitative variables were checked for normality distribution applying the Kolmogorov–Smirnov test. If the variable departed significantly from normality, it was stated as median and interquartile range (Q1–Q3). Categorical and normally distributed continuous variables were expressed as frequencies (percentages) and mean ± SD, respectively. The clinical and pathologic features at the time of diagnosis were compared between various DLBCL subgroups by using unpaired *t*-test when data were normally distributed. Otherwise, Mann–Whitney U test was conducted. The chi-squared test was used to evaluate whether a significant difference was present between the categorical variables. Kaplan–Meier estimator analyzed overall survival (OS) (calculated from the date of diagnosis of DLBCL until death or last follow-up) and progression free survival (PFS) (time from starting treatment to disease progression or death) curves of the various groups, and they were compared by log-rank test. Univariate and/or multivariate Cox regression analyses was used to assess the influence of variables on survival. A difference with a *p*-value of <0.05 was considered statistically significant.

### 2.5. Ethical Considerations

All procedures performed in the current study were performed in accordance with the 1964 Helsinki declaration and its later amendments. Approval by the Institutional Review Board (IRB) of Mount Sinai Medical Center of Florida was obtained before the study. Chart review was carried out by CITI (Collaborative Institutional Training Initiative) certified physicians.

## 3. Results

### 3.1. Clinicopathological Parameters

In total, 177 patients met the inclusion criteria. The clinical and pathological parameters are summarized in Table 1. The mean age was 73.20 ± 13.68 years. Our cohort included 91 men (51.4%) and 86 women (48.6%). In all, 38 (21.5%) patients had primary nodal lymphomas, while 139 (78.5%) had extranodal involvement. This high percentage of extranodal involvement could be explained by the rising incidence of extranodal disease, including the gastrointestinal tract, tonsils, lung, liver, spleen, bone, and skin [21,22]. At the time of presentation, a large proportion of patients had high stage disease (82.8% of 64 patients with stage 3 or higher) and high LDH level (64.2% of 137 patients with >225 U/L; reference range 135–225 U/L). Different treatment regimens were used in our cohort of patients depending on each patient’s medical condition. In our study, 69 patients were alive as of the last follow-up date (mean follow-up time 31.77 months, ranging from 1 to 114 months) while 24 patients died during follow-up (mean follow-up time 18.42 months, ranging from 0.75 to 88 months). The remaining 84 cases were lost to follow-up. The overall follow-up period ranged from 0.75 to 114 months, with a mean of 28.39 months (reflecting the overall survival, OS). The mean progression free survival (PFS) was 19.68 ± 23.55 months.

The widely accepted Hans algorithm [17] which utilizes CD10, BCL6, and MUM1 immunohistochemical stains to classify DLBCL was used to categorize our patients into GCB subtype (85 out of 170 cases; 50%) and non-GCB subtype (85 out of 170 cases; 50%). Ki-67 proliferation index was high in 102/165 (61.8%) patients and low in 63/165 (38.2%) patients. Monoclonal B-cell population was identified in 68/92 (73.9%) patients based on flow cytometry results. By immunohistochemistry, 39/64 cases (60.9%) expressed MYC, and 62/64 cases (96.9%) expressed BCL2. MYC/BCL2 double expression was identified in 38/64 (59.4%) patients. Of the total 177 cases, 83 showed positive expression of p53 (46.9%) and 79 showed positive expression of p63 (44.6%). In addition, 46 cases showed p53/p63 co-positivity (26%).

### 3.2. Correlation between p63 Positivity and Clinicopathological Parameters

We assessed the correlation of clinicopathological parameters with p63 expression among DLBCL patients (Table 2). A significant correlation was found between p63 positivity and p53 expression (*p* = 0.007), p53/p63 co-positivity (*p* < 0.001), high Ki-67 proliferation index (*p* = 0.002), MYC expression (*p* = 0.042), and MYC/BCL2 double expression (*p* = 0.025). Compared to p63− patients, the p63+ group had significantly higher mean p53 percentage (31.91 ± 26.36% vs. 22.33 ± 24.78%; *p* = 0.004). 

After stratifying patients into GCB and non-GCB, both subgroups showed significant correlation between p63+ and p53/p63 co-positivity (*p* < 0.001). The non-GCB group demonstrated significant correlation between p63+ and p53 positivity (*p* = 0.01) and the GCB group revealed significant correlation between p63+ and high Ki-67 proliferation index (*p* = 0.034). No statistical significance was seen with the other studied parameters such as age, gender, LDH level, clinical stage, survival, OS and PFS duration, presence of extranodal disease, or BCL2 expression. It was noted, however, that among the non-CGB group, there was a trend among p63+ patients to have MYC/BCL2 double expression. As for the mean OS and PFS, p63+ expression showed a trend of lower OS as well as PFS compared to the p63- group regardless of the GCB subtype, though it did not meet statistical significance (Table 2).

Since DLBCL patients with MYC/BCL2 double expression have been shown to have poor outcomes [23], we sought to further stratify our cohort of patients into double expressors and non-double expressors. Both groups showed significant correlation between p63 positivity and p53 expression (*p* < 0.001) as well as p53/p63 co-positivity (*p* = 0.002 and *p* < 0.001, respectively). In addition, among both subgroups of DLBCL patients, there was a trend of lower OS and PFS among p63+ patients compared to p63- patients, which was more evident among the non-double expressors (Table 3).

### 3.3. Correlation between p53 Positivity and Clinicopathological Parameters

We also assessed the correlation of clinicopathological parameters with p53 expression among DLBCL patients (Table 3). A significant correlation was found between p53 positivity and p63 expression (*p* = 0.007), p53/p63 co-positivity (*p* < 0.001), and high Ki-67 proliferation index (*p* = 0.005). In comparison to p53– patients, the p53+ group had significantly higher mean p63 percentage (33.22 ± 30.15% vs. 20.17 ± 25.08%; *p* = 0.008). In addition, there was a trend toward a higher LDH level (632.69 ± 803.33 U/L vs. 480.63 ± 414.32 U/L; *p* = 0.555) among p53+ patients. 

Similar to the p63 positive cases, the p53 positive group, after stratifying patients into GCB and non-GCB subgroups, showed significant correlation between p53+ and p53/p63 co-positivity (*p* < 0.001) in both subgroups, while the non-GCB group demonstrated significant correlation between p53+ and p63 positivity (*p* = 0.01). The GCB group revealed significant correlation between p53+ and high mean p63 percentage (36.75 ± 26.19% vs. 25.12 ± 28.08%; *p* = 0.019). No statistical significance was seen between p53 positivity and age, gender, LDH level, clinical stage, survival, OS and PFS duration, presence of extranodal disease, flow cytometry results, MYC, BCL2 expression, and MYC/BCL2 double expression (Table 4). In all patients, regardless of the GCB subgrouping, there was a trend among p53+ patients to have MYC/BCL2 double expression, positive MYC expression, and lower OS and PFS compared to the p53− group (Table 5).

### 3.4. Multivariate Survival Analysis

We performed multivariate Cox survival analyses to assess the prognostic significance of p53 and p63 expression (Table 6). When taking p53 and p63 as the only predictors, no statistical significance was demonstrated although differential trends were seen with p53+ and p63+ on OS and PFS (Table 6). 

### 3.5. Survival Curves

**Figure 2:**
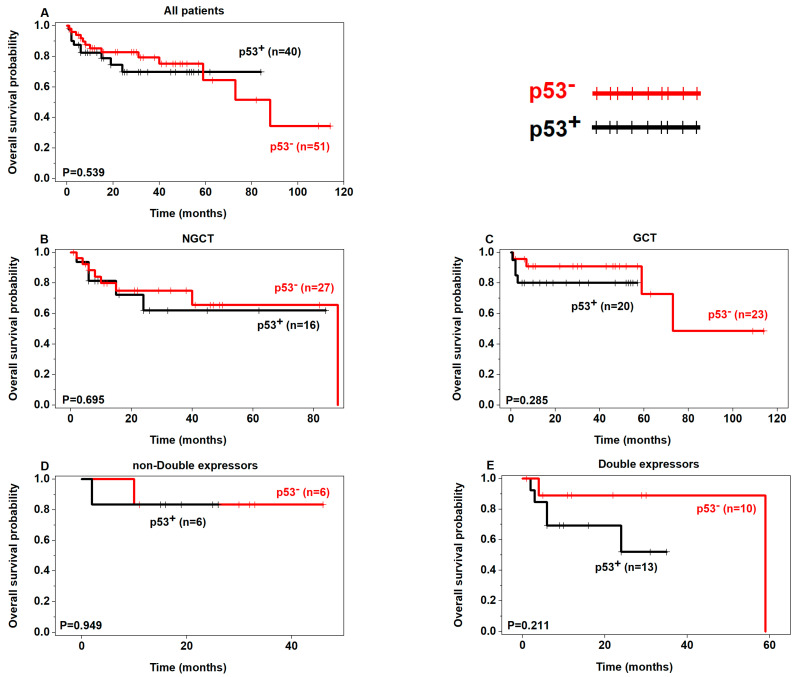
Prognostic analysis of p53 expression in DLBCL (OS) (p53+ in black, p53– in red).

**Figure 3:**
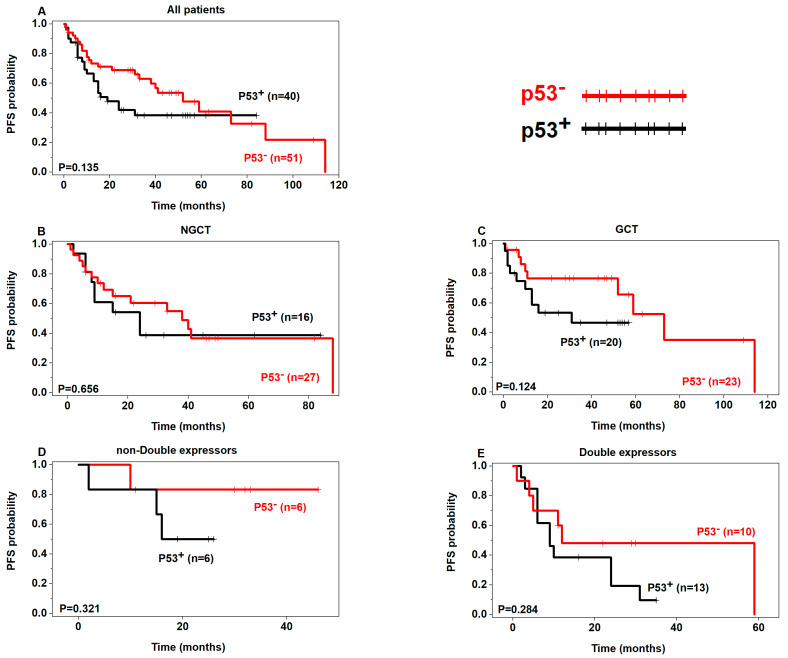
Prognostic analysis of p53 expression in DLBCL (PFS) (p53+ in black, p53– in red).

**Figure 4:**
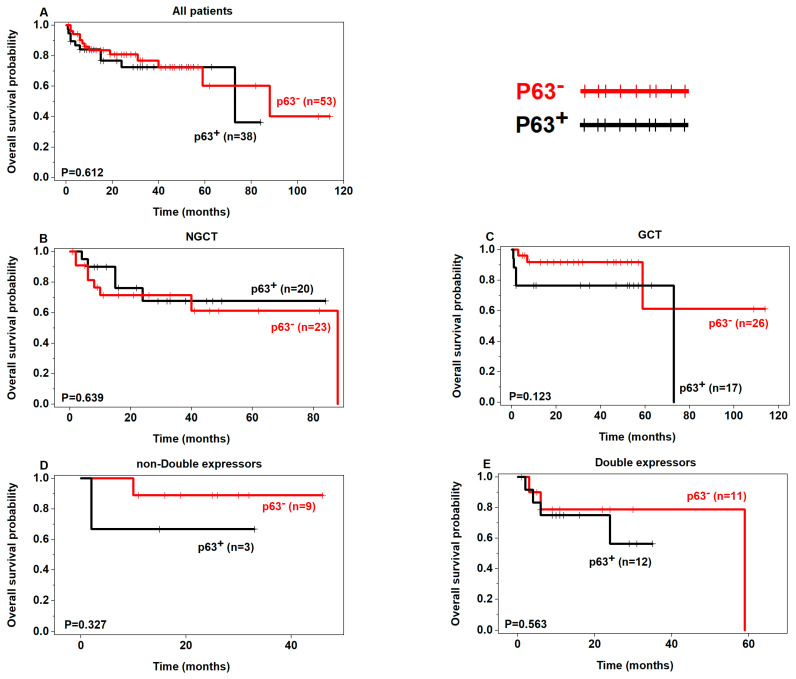
Prognostic analysis of p63 expression in DLBCL (OS) (p63+ in black, p63− in red).

**Figure 5:**
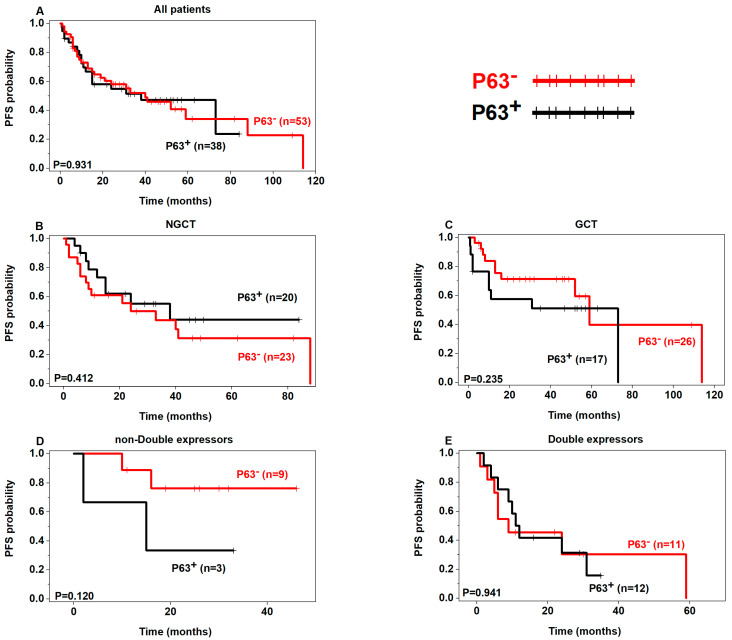
Prognostic analysis of p63 expression in DLBCL (PFS) (p63+ in black, p63− in red).

Although not statistically significant, a tendency of poor OS was seen in p53+ patients in the non-GCB, GCB, and double expressors subgroups (Figure 2) and poor PFS in p53+ patients regardless of the subgroups (Figure 3). In addition, there was a tendency of poor OS and PFS in p63+ patients in the non-double expressors and double expressors subgroups (Figure 4 and Figure 5). 

## 4. Discussion

We found that p53+ and p63+ have a propensity for poor prognosis in DLBCL as reflected in the OS and PFS. It has been postulated that the tumor suppressor p53 and its oncogenic sibling p63 have opposing fates in tumor development and progression [24]. In lymphoma, studies have evaluated the dual role of these biomarkers in predicting prognosis [15,25]. Some studies have shown that these two biomarkers might be useful in predicting a better prognosis while others revealed a poor prognostic value in DLBCL patients. Hedvat et al. showed that high p63 expression is correlated with high Ki-67 but not with OS [26]. Their cutoff for high p63 expression, however, was >10%, which is lower than ours. Compared to their results, we found a significant correlation with high Ki-67 (≥80%) in GCB p63+ and GCB p53+. Similarly, Robson et al. demonstrated a positive correlation between p63 expression and Ki-67, but no association with survival status [27].

Hallack et al. [16] concluded that patients with high p63 expression in high-risk DLBCL have better disease-free survival. In their study, p63 expression was considered high if >50% of cells stained positive. However, our patient cohort is larger (177 versus 73 cases) and the age range is wider [16]. We postulate that having a younger population in other studies compared to ours could explain the better disease-free survival reported. Xu-Monette et al. [15] showed that p63 expression depicted significantly better clinical outcomes, including PFS and OS, in high-risk DLBCL groups. In our multivariate survival analysis, non-GCB p63+ cases showed a trend for better OS and PFS (Fig 3 and 5). In their multivariate survival analysis, Xu-Monette et al. showed that mutated p53 overexpression was an independent prognostic factor for poor PFS [15]. Similarly, p53+ expression was associated with poor OS and PFS in our multivariate survival analysis, in the GCB, non-GCB, and double expressor subgroups.

Park et al. [28] concluded that p63 overexpression (>30%) signified poor survival in DLBCL, whereas p53 expression showed no correlation with survival. Similarly, Fukushima et al. [13] showed a trend of poor OS in DLBCL p63+ with a cut-off of ≥20%. Hu et al. [14] concluded that p63 and p53 co-expression had poor overall survival. They had a similar cohort as ours; however, their p63 expression was based on the Allred scoring system and the p53 cut-off value was >50% for overexpression. An interesting finding in our study was p53/p63 co-positivity being statistically significant in GCB and non-GCB subtypes. Hu et al. [14] and Xu-Monette et at. [15] assessed its prognostic implications in DLBCL as well showing improved survival. 

Limitations in our study include the relatively small number of patients in our cohort. In addition, cytogenetic studies and molecular ancillary tests were not included in the assessment. Loss of follow-up might have also potentially affected our results. We believe that the discrepancy in results between the studies could be due to the different criteria and cutoffs used to assess overexpression of these markers and the low sample sizes in different studies.

## 5. Conclusions

In conclusion, our results suggest that p63 and p53 might present potential additional biomarkers of prognosis in DLBCL. Integrating this to the different subgroups (GCB, non-GCB, double expressors and non-double expressors) would help to improve risk stratification and prognostic assessment in DLBCL. However, a larger study may need to be performed in order to better assess the prognostic significance of those two markers in DLBCL and other lymphomas as well.

## Figures and Tables

**Table 1:** Clinicopathological characteristics of patients (*N* = 177).

Clinicopathological Variables	Number of Patients (%)
Age (years)—Mean ± SD (*n* = 177)	73.20 ± 13.68
Age (years) (*n* = 177)	
<60	27 (15.3)
60–70<	32 (18.1)
70–80<	61 (34.5)
≥80	57 (32.2)
Gender (*n* = 177)	
Female	86 (48.6)
Male	91 (51.4)
p53 expression (*n* = 177)	
Negative	94 (53.1)
Positive	83 (46.9)
p63 expression (*n* = 177)	
Negative	98 (55.4)
Positive	79 (44.6)
p53/p63 co-positivity (*n* = 177)	
No	131 (74.0)
Yes	46 (26.0)
p53%—Mean ± SD (*n* = 177)	26.61 ± 25.87
p53%—Median (Q1–Q3)	18.39 (4.56–42.53)
p63%—Mean ± SD (*n* = 177)	26.29 ± 28.25
p63%—Median (Q1–Q3)	15.09 (2.07–46.02)
LDH (U/L)—Mean ± SD (*n* = 137)	552.77 ± 631.76
LDH (U/L)—Median (Q1–Q3)	370.00 (203.50–646.00)
LDH level (*n* = 137)	
High (>225 U/L)	88 (64.2)
Normal (135–225 U/L)	49 (35.8)
Clinical stage (*n* = 64)	
1	8 (12.5)
2	3 (4.7)
3	22 (34.4)
4	31 (48.4)
Clinical stage category, low vs. high (*n* = 64)	
Low (1–2)	11 (17.2)
High (3–4)	53 (82.8)
Response (*n* = 93)	
Recurrence	26 (28.0)
Remission	43 (46.2)
Death	24 (25.8)
Survival (*n* = 93)	
No	24 (25.8)
Yes	69 (74.2)
OS among all patients (months)—Mean ± SD (*n* = 91)	28.39 ± 24.96
OS among all patients (months)—Median (Q1–Q3)	22.00 (8.00–46.00)
Follow-up duration among alive patients (months)—Mean ± SD (*n* = 68)	31.77 ± 24.42
Follow-up duration among alive patients (months)—Median (Q1–Q3)	28.50 (11.00–47.00)
Follow-up duration among patients who died (months)—Mean ± SD (*n* = 23)	18.42 ± 24.36
Follow-up duration among patients who died (months)—Median (Q1–Q3)	7.00 (2.50–22.50)
PFS (months)—Mean ± SD (*n* = 48)	19.68 ± 23.55
PFS (months)—Median (Q1–Q3)	10.50 (6.00–24.00)
Subtype (*n* = 170)	
Non-GCB	85 (50.0)
GCB	85 (50.0)
Extranodal (*n* = 177)	
No	38 (21.5)
Yes	139 (78.5)
Ki-67 (*n* = 165)	
Low (<80%)	63 (38.2)
High (≥80%)	102 (61.8)
Flow cytometry (*n* = 92)	
Negative	24 (26.1)
Monoclonal B-cell population	68 (73.9)
MYC expression (*n* = 64)	
Negative	25 (39.1)
Positive	39 (60.9)
BCL2 expression (*n* = 64)	
Negative	2 (3.1)
Positive	62 (96.9)
Double expressor (*n* = 64)	
No	26 (40.6)
Yes	38 (59.4)

Abbreviations: GCB: germinal center B-cell like; LDH: lactate dehydrogenase; non-GCB: non-germinal center B-cell like; OS: overall survival; PFS: progression free survival; Q1: first quartile; Q3: third quartile.

**Table 2:** Correlation between p63 positivity and clinicopathological characteristics of patients stratified into GCB and non-GCB subgroups (*N* = 177).

Clinicopathological Variables	All Patients (*N* = 177)			GCB (*N* = 85)			Non-GCB (*N* = 85)		
	p63−	p63+	*p*-Value	p63−	p63+	*p*-Value	p63−	p63+	*p*-Value
Age (years)—Mean ± SD	74.08 ± 13.13	72.10 ± 14.33	0.340	72.08 ± 12.97	72.79 ± 13.56	0.809	76.85 ± 13.80	71.29 ± 14.99	0.080
Age (years)			0.830			0.883			0.474
<60	13 (13.3)	14 (17.7)		9 (17.3)	5 (15.2)		4 (10.0)	9 (20.0)	
60–70<	17 (17.3)	15 (19.0)		10 (19.2)	7 (21.2)		5 (12.5)	8 (17.8)	
70–80<	35 (35.7)	26 (32.9)		17 (32.7)	13 (39.4)		15 (37.5)	13 (28.9)	
≥80	33 (33.7)	24 (30.4)		16 (30.8)	8 (24.2)		16 (40.0)	15 (33.3)	
Gender			0.625			0.950			0.609
Female	46 (46.9)	40 (50.6)		24 (46.2)	15 (45.5)		20 (50.0)	25 (55.6)	
Male	52 (53.1)	39 (49.4)		28 (53.8)	18 (54.5)		20 (50.0)	20 (44.4)	
p53 expression			0.007			0.170			0.010
Negative	61 (62.2)	33 (41.8)		30 (57.7)	14 (42.4)		28 (70.0)	19 (42.2)	
Positive	37 (37.8)	46 (58.2)		22 (42.3)	19 (57.6)		12 (30.0)	26 (57.8)	
p63 expression			<0.001			<0.001			<0.001
Negative	98 (100.0)	0 (0.0)		52 (100.0)	0 (0.0)		40 (100.0)	0 (0.0)	
Positive	0 (0.0)	79 (100.0)		0 (0.0)	33 (100.0)		0 (0.0)	45 (100.0)	
p53/p63 co-positivity			<0.001			<0.001			<0.001
No	98 (100.0)	33 (41.8)		52 (100.0)	14 (42.4)		40 (100.0)	19 (42.2)	
Yes	0 (0.0)	46 (58.2)		0 (0.0)	19 (57.6)		0 (0.0)	26 (57.8)	
p53%—Mean ± SD	22.33 ± 24.78	31.91 ± 26.36	0.004 *	25.23 ± 26.90	31.01 ± 25.51	0.200 *	16.39 ± 18.66	31.91 ± 27.16	0.003 *
p53%—Median (Q1–Q3)	11.97 (3.55–33.75)	24.59 (7.24–51.79)		14.85 (3.35–36.62)	23.65 (8.00–50.89)		8.49 (3.61–25.06)	24.59 (7.15–53.45)	
p63%—Mean ± SD	5.33 ± 5.62	52.30 ± 22.91	<0.001 *	3.88 ± 4.44	53.82 ± 23.53	<0.001 *	7.27 ± 6.38	50.81 ± 22.74	<0.001 *
P63%—Median (Q1–Q3)	2.59 (0.96–8.52)	46.94 (32.28–69.05)		1.83 (0.76–6.50)	47.10 (32.46–77.00)		4.69 (1.84–13.88)	46.32 (31.73–63.84)	
LDH (U/L)—Mean ± SD	626.82 ± 720.03	442.38 ± 454.74	0.063 *	681.14 ± 763.68	524.48 ± 605.46	0.247 *	474.62 ± 451.82	394.19 ± 301.06	0.458 *
LDH (U/L)—Median (Q1–Q3)	400.00 (218.50–707.00)	305.00 (190.00–560.00)		404.00 (247.00–776.00)	360.00 (190.00–641.00)		370.50 (196.25–509.50)	303.00 (200.00–482.00)	
LDH level			0.116			0.254			0.417
High	57 (69.5)	31 (56.4)		32 (74.4)	14 (60.9)		22 (64.7)	17 (54.8)	
Normal	25 (30.5)	24 (43.6)		11 (25.6)	9 (39.1)		12 (35.3)	14 (45.2)	
Clinical stage			0.577			0.428			0.520
1	4 (11.1)	4 (14.3)		1 (6.3)	2 (18.2)		3 (17.6)	2 (11.8)	
2	1 (2.8)	2 (7.1)		1 (6.3)	0 (0.0)		0 (0.0)	2 (11.8)	
3	11 (30.6)	11 (39.3)		6 (37.5)	6 (54.5)		5 (29.4)	5 (29.4)	
4	20 (55.6)	11 (39.3)		8 (50.0)	3 (27.3)		9 (52.9)	8 (47.1)	
Clinical stage category, low vs. high			0.428			0.683			0.671
Low (1–2)	5 (13.9)	6 (21.4)		2 (12.5)	2 (18.2)		3 (17.6)	4 (23.5)	
High (3–4)	31 (86.1)	22 (78.6)		14 (87.5)	9 (81.8)		14 (82.4)	13 (76.5)	
Response			0.896			0.505			0.410
Recurrence	16 (29.6)	10 (25.6)		7 (25.9)	5 (27.8)		7 (30.4)	4 (20.0)	
Remission	24 (44.4)	19 (48.7)		16 (59.3)	8 (44.4)		8 (34.8)	11 (55.0)	
Death	14 (25.9)	10 (25.6)		4 (14.8)	5 (27.8)		8 (34.8)	5 (25.0)	
Survival			0.975			0.287			0.486
No	14 (25.9)	10 (25.6)		4 (14.8)	5 (27.8)		8 (34.8)	5 (25.0)	
Yes	40 (74.1)	29 (74.4)		23 (85.2)	13 (72.2)		15 (65.2)	15 (75.0)	
OS (months)—Mean ± SD	29.49 ± 26.65	26.86 ± 22.65	0.763 *	34.35 ± 28.99	26.69 ± 26.01	0.628 *	26.04 ± 25.35	25.05 ± 20.28	0.742 *
OS (months)—Median (Q1–Q3)	22.00 (8.00–46.00)	19.00 (7.50–47.00)		29.00 (11.75–49.75)	31.00 (2.00–54.00)		16.00 (6.00–41.00)	19.00 (8.25–36.75)	
PFS (months)—Mean ± SD	22.69 ± 26.77	15.09 ± 17.21	0.398 *	29.10 ± 35.69	15.64 ± 23.50	0.165 *	19.73 ± 23.36	14.56 ± 10.63	0.811 *
PFS (months)—Median (Q1–Q3)	13.00 (6.00–31.00)	10.00 (5.00–15.00)		13.00 (7.00–52.00)	10.00 (2.00–11.00)		9.00 (5.50–28.50)	12.00 (8.00–15.00)	
Subtype			0.065			-			-
Non-GCB	40 (43.5)	45 (57.7)		-	-		-	-	
GCB	52 (56.5)	33 (42.3)		-	-		-	-	
Extranodal			0.453			0.739			0.277
No	19 (19.4)	19 (24.1)		11 (21.2)	8 (24.2)		6 (15.0)	11 (24.4)	
Yes	79 (80.6)	60 (75.9)		41 (78.8)	25 (75.8)		34 (85.0)	34 (75.6)	
Ki-67			0.002			0.034			0.057
<80%	44 (48.9)	19 (25.3)		27 (52.9)	9 (29.0)		16 (43.2)	10 (23.3)	
≥80%	46 (51.1)	56 (74.7)		24 (47.1)	22 (71.0)		21 (56.8)	33 (76.7)	
Flow cytometry			0.283			0.835			0.356
Negative	10 (21.3)	14 (31.1)		5 (17.2)	3 (15.0)		5 (31.3)	11 (45.8)	
Monoclonal B-cell population	37 (78.7)	31 (68.9)		24 (82.8)	17 (85.0)		11 (68.8)	13 (54.2)	
MYC expression			0.042			0.268			0.109
Negative	18 (50.0)	7 (25.0)		12 (66.7)	4 (44.4)		6 (33.3)	2 (11.1)	
Positive	18 (50.0)	21 (75.0)		6 (33.3)	5 (55.6)		12 (66.7)	16 (88.9)	
BCL2 expression			0.205			0.471			0.310
Negative	2 (5.6)	0 (0.0)		1 (5.6)	0 (0.0)		1 (5.6)	0 (0.0)	
Positive	34 (94.4)	28 (100.0)		17 (94.4)	9 (100.0)		17 (94.4)	18 (100.0)	
Double expressor			0.025			0.268			0.054
No	19 (52.8)	7 (25.0)		12 (66.7)	4 (44.4)		7 (38.9)	2 (11.1)	
Yes	17 (47.2)	21 (75.0)		6 (33.3)	5 (55.6)		11 (61.1)	16 (88.9)	

Abbreviations: GCB: germinal center B-cell like; LDH: lactate dehydrogenase; non-GCB: non-germinal center B-cell like; OS: overall survival; PFS: progression free survival; Q_1_: first quartile; Q_3_: third quartile. * Mann–Whitney *U* Test.

**Table 3:** Correlation between p63 positivity and clinicopathological characteristics of patients stratified into non-double expressors and double expressors (*N* = 64).

Clinicopathological Variables	Non-Double Expressors (*N* = 26)			Double Expressors (*N* = 38)		
	p63−	p63+	*p*-Value	p63−	p63+	*p*-Value
Age (years)—Mean ± SD	73.53 ± 9.51	69.43 ± 14.59	0.408	75.18 ± 9.18	73.95 ± 14.18	0.760
Age (years)			0.959			0.813
<60	2 (10.5)	1 (14.3)		1 (5.9)	1 (4.8)	
60–70<	4 (21.1)	2 (28.6)		3 (17.6)	6 (28.6)	
70–80<	7 (36.8)	2 (28.6)		7 (41.2)	6 (28.6)	
≥80	6 (31.6)	2 (28.6)		6 (35.3)	8 (38.1)	
Gender			0.973			0.492
Female	11 (57.9)	4 (57.1)		7 (41.2)	11 (52.4)	
Male	8 (42.1)	3 (42.9)		10 (58.8)	10 (47.6)	
p53 expression			0.780			0.116
Negative	12 (63.2)	4 (57.1)		10 (58.8)	7 (33.3)	
Positive	7 (36.8)	3 (42.9)		7 (41.2)	14 (66.7)	
p53/p63 co-positivity			0.002			<0.001
No	19 (100.0)	4 (57.1)		17 (100.0)	7 (33.3)	
Yes	0 (0.0)	3 (42.9)		0 (0.0)	14 (66.7)	
p53%—Mean ± SD	16.17 ± 16.09	17.06 ± 21.83	0.977 *	24.91 ± 24.51	38.05 ± 28.10	0.154 *
p53%—Median (Q1–Q3)	10.99 (2.36–25.83)	4.95 (1.55–24.59)		16.20 (6.49–36.27)	32.52 (13.87–67.05)	
LDH—Mean ± SD	364.08 ± 247.38	254.50 ± 160.31	0.571 *	714.50 ± 1021.22	461.08 ± 395.60	0.738 *
LDH—Median (Q1–Q3)	274.00 (192.50–447.00)	279.00 (89.50–395.00)		331.50 (222.25–531.50)	289.50 (174.00–710.75)	
LDH level (U/L)			0.893			0.756
High	7 (53.8)	2 (50.0)		9 (64.3)	7 (58.3)	
Normal	6 (46.2)	2 (50.0)		5 (35.7)	5 (41.7)	
Clinical stage			0.788			0.485
1	1 (11.1)	0 (0.0)		1 (14.3)	1 (12.5)	
2	-	-		0 (0.0)	1 (12.5)	
3	2 (22.2)	0 (0.0)		1 (14.3)	3 (37.5)	
4	6 (66.7)	1 (100.0)		5 (71.4)	3 (37.5)	
Clinical stage category, low vs. high			0.725			0.605
Low (1–2)	1 (11.1)	0 (0.0)		1 (14.3)	2 (25.0)	
High (3–4)	8 (88.9)	1 (100.0)		6 (85.7)	6 (75.0)	
Response			0.368			0.951
Recurrence	1 (11.1)	1 (33.3)		5 (45.5)	5 (41.7)	
Remission	7 (77.8)	1 (33.3)		3 (27.3)	3 (25.0)	
Death	1 (11.1)	1 (33.3)		3 (27.3)	4 (33.3)	
Survival			0.371			0.752
No	1 (11.1)	1 (33.3)		3 (27.3)	4 (33.3)	
Yes	8 (88.9)	2 (66.7)		8 (72.7)	8 (66.7)	
OS (months)—Mean ± SD	23.89 ± 11.42	16.67 ± 15.57	0.518 *	16.00 ± 17.13	15.75 ± 11.22	0.517 *
OS (months)—Median (Q1–Q3)	25.00 (13.50–31.00)	15.00 (8.50–24.00)		9.00 (5.00–24.00)	11.50 (6.75–27.75)	
PFS (months)—Mean ± SD	13.00 ± 4.24	8.50 ± 9.19	0.439 *	14.12 ± 19.45	12.11 ± 9.48	0.440 *
PFS (months)—Median (Q1–Q3)	13.00 (10.00–16.00)	8.50 (2.00–15.00)		6.00 (4.00–16.50)	10.00 (6.00–12.00)	
Subtype			0.876			0.438
Non-GCB	12 (63.2)	4 (66.7)		11 (64.7)	16 (76.2)	
GCB	7 (36.8)	2 (33.3)		6 (35.3)	5 (23.8)	
Extranodal			0.908			0.726
No	5 (26.3)	2 (28.6)		4 (23.5)	6 (28.6)	
Yes	14 (73.7)	5 (71.4)		13 (76.5)	15 (71.4)	
Ki-67			0.780			0.495
<80%	12 (63.2)	4 (57.1)		6 (35.3)	5 (25.0)	
≥80%	7 (36.8)	3 (42.9)		11 (64.7)	15 (75.0)	
Flow cytometry			0.398			0.013
Negative	5 (55.6)	2 (33.3)		0 (0.0)	5 (50.0)	
Monoclonal B-cell population	4 (44.4)	4 (66.7)		9 (100.0)	5 (50.0)	
MYC expression			0.536			-
Negative	18 (94.7)	7 (100.0)		-	-	
Positive	1 (5.3)	0 (0.0)		17 (100.0)	21 (100.0)	
BCL2 expression			0.372			-
Negative	2 (10.5)	0 (0.0)		-	-	
Positive	17 (89.5)	7 (100.0)		17 (100.0)	21 (100.0)	

Abbreviations: GCB: germinal center B-cell like; LDH: lactate dehydrogenase; non-GCB: non-germinal center B-cell like; OS: overall survival; PFS: progression free survival; Q_1_: first quartile; Q_3_: third quartile. * Mann–Whitney *U* Test.

**Table 4:** Correlation between p53 positivity and clinicopathological characteristics of patients stratified into non-double expressors and double expressors (*N* = 64).

Clinicopathological Variables	Non-Double Expressors (*N* = 26)			Double Expressors (*N* = 38)		
	p53−	p53+	*p*-Value	p53−	p53+	*p*-Value
Age (years)—Mean ± SD	70.06 ± 11.61	76.20 ± 9.07	0.196	74.47 ± 10.16	74.52 ± 13.65	0.989
Age (years)			0.610			0.652
<60	2 (12.5)	1 (10.0)		1 (5.9)	1 (4.8)	
60–70<	5 (31.3)	1 (10.0)		5 (29.4)	4 (19.0)	
70–80<	5 (31.3)	4 (40.0)		4 (23.5)	9 (42.9)	
≥80	4 (25.0)	4 (40.0)		7 (41.2)	7 (33.3)	
Gender			0.315			0.973
Female	8 (50.0)	7 (70.0)		8 (47.1)	10 (47.6)	
Male	8 (50.0)	3 (30.0)		9 (52.9)	11 (52.4)	
p63 expression			0.780			0.116
Negative	12 (75.0)	7 (70.0)		10 (58.8)	7 (33.3)	
Positive	4 (25.0)	3 (30.0)		7 (41.2)	14 (66.7)	
p53/p63 co-positivity			0.020			<0.001
No	16 (100.0)	7 (70.0)		17 (100.0)	7 (33.3)	
Yes	0 (0.0)	3 (30.0)		0 (0.0)	14 (66.7)	
p63%—Mean ± SD	19.09 ± 24.17	24.03 ± 30.20	0.874 *	25.23 ± 31.41	38.05 ± 30.42	0.223 *
p63%—Median (Q1–Q3)	8.93 (2.64–25.74)	14.33 (1.49–37.60)		13.52 (3.05–31.30)	37.53 (4.72–62.06)	
LDH—Mean ± SD	355.40 ± 276.56	313.86 ± 159.53	0.770 *	508.75 ± 626.56	673.64 ± 928.19	0.571 *
LDH—Median (Q1–Q3)	230.50 (183.75–435.00)	334.00 (246.00–370.00)		246.00 (207.25–542.50)	350.00 (207.25–664.25)	
LDH level			0.201			0.263
High	4 (40.0)	5 (71.4)		6 (50.0)	10 (71.4)	
Normal	6 (60.0)	2 (28.6)		6 (50.0)	4 (28.6)	
Clinical stage			0.240			0.175
1	1 (16.7)	0 (0.0)		0 (0.0)	2 (28.6)	
2	-	-		0 (0.0)	1 (14.3)	
3	2 (33.3)	0 (0.0)		2 (25.0)	2 (28.6)	
4	3 (50.0)	4 (100.0)		6 (75.0)	2 (28.6)	
Clinical stage category, low vs. high			0.389			0.038
Low (1–2)	1 (16.7)	0 (0.0)		0 (0.0)	3 (42.9)	
High (3–4)	5 (83.3)	4 (100.0)		8 (100.0)	4 (57.1)	
Response			0.287			0.369
Recurrence	0 (0.0)	2 (33.3)		4 (40.0)	6 (46.2)	
Remission	5 (83.3)	3 (50.0)		4 (40.0)	2 (15.4)	
Death	1 (16.7)	1 (16.7)		2 (20.0)	5 (38.5)	
Survival			1.000			0.340
No	1 (16.7)	1 (16.7)		2 (20.0)	5 (38.5)	
Yes	5 (83.3)	5 (83.3)		8 (80.0)	8 (61.5)	
OS (months)—Mean ± SD	27.00 ± 13.97	17.17 ± 8.70	0.174	18.40 ± 17.47	13.92 ± 11.02	0.460
OS (months)—Median (Q1–Q3)	31.00 (10.75–36.25)	17.50 (11.75–25.25)		11.50 (4.75–29.25)	9.00 (6.00–24.00)	
PFS (months)—Mean ± SD	-	11.00 ± 7.81	-	15.33 ± 21.80	11.82 ± 9.80	0.840 *
PFS (months)—Median (Q1–Q3)	-	15.00 (8.50–15.50)		8.00 (4.00–12.00)	9.00 (6.00–17.00)	
Subtype			0.835			0.955
Non-GCB	6 (37.5)	3 (33.3)		12 (70.6)	15 (71.4)	
GCB	10 (62.5)	6 (66.7)		5 (29.4)	6 (28.6)	
Extranodal			0.124			0.061
No	6 (37.5)	1 (10.0)		7 (41.2)	3 (14.3)	
Yes	10 (62.5)	9 (90.0)		10 (58.8)	18 (85.7)	
Ki-67			0.074			0.495
<80%	12 (75.0)	4 (40.0)		6 (35.3)	5 (25.0)	
≥80%	4 (25.0)	6 (60.0)		11 (64.7)	15 (75.0)	
Flow cytometry			0.143			0.345
Negative	6 (60.0)	1 (20.0)		2 (18.2)	3 (37.5)	
Monoclonal B-cell population	4 (40.0)	4 (80.0)		9 (81.8)	5 (62.5)	
MYC expression			0.197			-
Negative	16 (100.0)	9 (90.0)		-	-	
Positive	0 (0.0)	1 (10.0)		17 (100.0)	21 (100.0)	
BCL2 expression			0.727			-
Negative	1 (6.3)	1 (10.0)		-	-	
Positive	15 (93.8)	9 (90.0)		17 (100.0)	21 (100.0)	

Abbreviations: GCB: germinal center B-cell like; LDH: lactate dehydrogenase; non-GCB: non-germinal center B-cell like; OS: overall survival; PFS: progression free survival; Q_1_: first quartile; Q_3_: third quartile. * Mann–Whitney *U* Test.

**Table 5:** Correlation between p53 positivity and clinicopathological characteristics of patients stratified into GCB and non-GCB subgroups (*N* = 177).

Clinicopathological Variables	All Patients (*N* = 177)			GCB (*N* = 85)			Non-GCB (*N* = 85)		
	p53−	p53+	*p*-Value	p53−	p53+	*p*-Value	p53−	p53+	*p*-Value
Age (years)—Mean ± SD	73.31 ± 13.80	73.07 ± 13.62	0.909	72.11 ± 13.55	72.61 ± 12.82	0.863	74.23 ± 14.49	73.50 ± 14.99	0.820
Age (years)			0.509			0.461			0.599
<60	14 (14.9)	13 (15.7)		8 (18.2)	6 (14.6)		6 (12.8)	7 (18.4)	
60–70<	19 (20.2)	13 (15.7)		10 (22.7)	7 (17.1)		9 (19.1)	4 (10.5)	
70–80<	28 (29.8)	33 (39.8)		12 (27.3)	18 (43.9)		14 (29.8)	14 (36.8)	
≥80	33 (35.1)	24 (28.9)		14 (31.8)	10 (24.4)		18 (38.3)	13 (34.2)	
Gender			0.192			0.221			0.959
Female	50 (53.2)	36 (43.4)		23 (52.3)	16 (39.0)		25 (53.2)	20 (52.6)	
Male	44 (46.8)	47 (56.6)		21 (47.7)	25 (61.0)		22 (46.8)	18 (47.4)	
p53 expression			<0.001			<0.001			<0.001
Negative	94 (100.0)	0 (0.0)		44 (100.0)	0 (0.0)		47 (100.0)	0 (0.0)	
Positive	0 (0.0)	83 (100.0)		0 (0.0)	41 (100.0)		0 (0.0)	38 (100.0)	
p63 expression			0.007			0.170			0.010
Negative	61 (64.9)	37 (44.6)		30 (68.2)	22 (53.7)		28 (59.6)	12 (31.6)	
Positive	33 (35.1)	46 (55.4)		14 (31.8)	19 (46.3)		19 (40.4)	26 (68.4)	
p53/p63 co-positivity			<0.001			<0.001			<0.001
No	94 (100.0)	37 (44.6)		44 (100.0)	22 (53.7)		47 (100.0)	12 (31.6)	
Yes	0 (0.0)	46 (55.4)		0 (0.0)	19 (46.3)		0 (0.0)	26 (68.4)	
p53%—Mean ± SD	6.86 ± 5.76	48.97 ± 21.11	<0.001 *	7.32 ± 6.32	49.10 ± 22.16	<0.001 *	6.52 ± 5.31	46.97 ± 20.44	<0.001 *
p53%—Median (Q1–Q3)	4.91 (1.94–10.99)	45.29 (30.50–68.28)		4.92 (1.99–12.88)	46.68 (31.06–66.89)		4.85 (1.63–9.71)	41.35 (28.34–68.53)	
p63%—Mean ± SD	20.17 ± 25.08	33.22 ± 30.15	0.008 *	15.68 ± 21.38	31.42 ± 33.22	0.127 *	25.12 ± 28.08	36.75 ± 26.19	0.019 *
P63%—Median (Q1–Q3)	8.93 (2.14–28.47)	29.46 (1.94–55.52)		6.49 (1.34–25.65)	15.94 (1.44–53.65)		15.09 (3.25–38.24)	35.33 (13.78–56.25)	
LDH (U/L)—Mean ± SD	480.63 ± 414.32	632.69 ± 803.33	0.555 *	484.94 ± 378.43	777.00 ± 929.71	0.270 *	469.75 ± 450.78	394.69 ± 290.20	0.697 *
LDH (U/L)—Median (Q1–Q3)	331.00 (197.00–688.50)	374.00 (215.00–611.00)		279.00 (195.25–773.00)	427.00 (256.75–719.75)		361.50 (193.25–520.50)	305.00 (199.50–498.00)	
LDH level			0.246			0.148			0.760
High	43 (59.7)	45 (69.2)		21 (61.8)	25 (78.1)		21 (58.3)	18 (62.1)	
Normal	29 (40.3)	20 (30.8)		13 (38.2)	7 (21.9)		15 (41.7)	11 (37.9)	
Clinical stage			0.103			0.247			0.302
1	3 (8.6)	5 (17.2)		1 (6.7)	2 (16.7)		2 (10.5)	3 (20.0)	
2	0 (0.0)	3 (10.3)		0 (0.0)	1 (8.3)		0 (0.0)	2 (13.3)	
3	15 (42.9)	7 (24.1)		9 (60.0)	3 (25.0)		6 (31.6)	4 (26.7)	
4	17 (48.6)	14 (48.3)		5 (33.3)	6 (50.0)		11 (57.9)	6 (40.0)	
Clinical stage category, low vs. high			0.045			0.183			0.102
Low (1–2)	3 (8.6)	8 (27.6)		1 (6.7)	3 (25.0)		2 (10.5)	5 (33.3)	
High (3–4)	32 (91.4)	21 (72.4)		14 (93.3)	9 (75.0)		17 (89.5)	10 (66.7)	
Response			0.684			0.784			0.993
Recurrence	13 (25.0)	13 (31.7)		6 (25.0)	6 (28.6)		7 (25.9)	4 (25.0)	
Remission	26 (50.0)	17 (41.5)		14 (58.3)	10 (47.6)		12 (44.4)	7 (43.8)	
Death	13 (25.0)	11 (26.8)		4 (16.7)	5 (23.8)		8 (29.6)	5 (31.3)	
Survival			0.841			0.550			0.911
No	13 (25.0)	11 (26.8)		4 (16.7)	5 (23.8)		8 (29.6)	5 (31.3)	
Yes	39 (75.0)	30 (73.2)		20 (83.3)	16 (76.2)		19 (70.4)	11 (68.8)	
OS (months)—Mean ± SD	32.47 ± 27.33	23.19 ± 20.74	0.138 *	39.09 ± 31.27	24.94 ± 21.05	0.169 *	26.89 ± 23.20	23.37 ± 22.88	0.580 *
OS (months)—Median (Q1–Q3)	30.00 (10.00–47.00)	15.50 (6.00–34.25)		32.00 (10.00–57.00)	17.50 (5.25–50.75)		21.00 (8.00–41.00)	15.50 (6.50–30.50)	
PFS (months)—Mean ± SD	27.60 ± 29.84	11.08 ± 8.01	0.103 *	37.22 ± 39.33	9.67 ± 9.26	0.153 *	21.60 ± 23.32	11.44 ± 7.91	0.511 *
PFS (months)—Median (Q1–Q3)	12.00 (7.00–40.00)	9.00 (6.00–15.00)		11.00 (8.00–59.00)	8.00 (2.00–13.00)		12.00 (5.50–35.50)	9.00 (6.00–15.00)	
Subtype			0.645			-			-
Non-GCB	47 (51.6)	38 (48.1)		-	-		-	-	
GCB	44 (48.4)	41 (51.9)		-	-		-	-	
Extranodal			0.301			0.099			0.827
No	23 (24.5)	15 (18.1)		13 (29.5)	6 (14.6)		9 (19.1)	8 (21.1)	
Yes	71 (75.5)	68 (81.9)		31 (70.5)	35 (85.4)		38 (80.9)	30 (78.9)	
Ki-67			0.005			0.006			0.148
<80%	42 (48.3)	21 (26.9)		25 (58.1)	11 (28.2)		17 (39.5)	9 (24.3)	
≥80%	45 (51.7)	57 (73.1)		18 (41.9)	28 (71.8)		26 (60.5)	28 (75.7)	
Flow cytometry			0.600			0.751			0.343
Negative	13 (24.1)	11 (28.9)		4 (14.8)	4 (18.2)		9 (34.6)	7 (50.0)	
Monoclonal B-cell population	41 (75.9)	27 (71.1)		23 (85.2)	18 (81.8)		17 (65.4)	7 (50.0)	
MYC expression			0.111			0.381			0.109
Negative	16 (48.5)	9 (29.0)		10 (66.7)	6 (50.0)		6 (33.3)	2 (11.1)	
Positive	17 (51.5)	22 (71.0)		5 (33.3)	6 (50.0)		12 (66.7)	16 (88.9)	
BCL2 expression			0.964			0.362			0.310
Negative	1 (3.0)	1 (3.2)		1 (6.7)	0 (0.0)		0 (0.0)	1 (5.6)	
Positive	32 (97.0)	30 (96.8)		14 (93.3)	12 (100.0)		18 (100.0)	17 (94.4)	
Double expressor			0.187			0.381			0.248
No	16 (48.5)	10 (32.3)		10 (66.7)	6 (50.0)		6 (33.3)	3 (16.7)	
Yes	17 (51.5)	21 (67.7)		5 (33.6)	6 (50.0)		12 (66.7)	15 (83.3)	

Abbreviations: GCB: germinal center B-cell like; LDH: lactate dehydrogenase; non-GCB: non-germinal center B-cell like; OS: overall survival; PFS: progression free survival; Q_1_: first quartile; Q_3_: third quartile. * Mann-Whitney *U* Test.

**Table 6:** Multivariate survival analysis with only p53 and p63 as predictors.

	OS			PFS		
Variable	HR	95% CI	*p*-Value	HR	95% CI	*p*-Value
All patients						
p53 expression						
Negative	1	-	-	1	-	-
Positive	1.250	0.519–3.007	0.619	1.586	0.867–2.903	0.135
p63 expression						
Negative	1	-	-	1	-	-
Positive	1.174	0.492–2.800	0.718	0.909	0.494–1.671	0.759
GCB						
p53 expression						
Negative	1	-	-	1	-	-
Positive	1.995	0.356–11.186	0.432	1.941	0.681–5.533	0.215
p63 expression						
Negative	1	-	-	1	-	-
Positive	2.628	0.613–11.271	0.193	1.478	0.564–3.869	0.427
Non-GCB						
p53 expression						
Negative	1	-	-	1	-	-
Positive	1.375	0.418–4.526	0.601	1.344	0.563–3.207	0.505
p63 expression						
Negative	1	-	-	1	-	-
Positive	0.705	0.214–2.318	0.564	0.659	0.277–1.568	0.346
Double expressors						
p53 expression						
Negative	1	-	-	1	-	-
Positive	3.358	0.380–29.696	0.276	1.890	0.617–5.792	0.265
p63 expression						
Negative	1	-	-	1	-	-
Positive	1.314	0.234–7.382	0.757	0.791	0.277–2.256	0.660
Non-double expressors						
p53 expression						
Negative	1	-	-	1	-	-
Positive	0.928	0.056–15.458	0.959	3.376	0.332–34.335	0.304
p63 expression						
Negative	1	-	-	1	-	-
Positive	3.713	0.223–61.833	0.361	4.882	0.600–39.757	0.138

Abbreviations: GCB: germinal center B-cell like; non-GCB: non-germinal center B-cell like; OS: overall survival; PFS: progression free survival.

## Data Availability

The data presented in this study are available in this article.
